# Three-dimensional analysis reveals two major architectural subgroups of prostate cancer growth patterns

**DOI:** 10.1038/s41379-019-0221-0

**Published:** 2019-02-08

**Authors:** Esther I. Verhoef, Wiggert A. van Cappellen, Johan A. Slotman, Gert-Jan Kremers, Patricia C. Ewing-Graham, Adriaan B. Houtsmuller, Martin E. van Royen, Geert J. L. H. van Leenders

**Affiliations:** 1 Department of Pathology, University Medical Center Rotterdam, Rotterdam, The Netherlands; 2 Department of Optical Imaging Center, Erasmus MC, University Medical Center Rotterdam, Rotterdam, The Netherlands

**Keywords:** Translational research, Prostate

## Abstract

The Gleason score is one of the most important parameters for therapeutic decision-making in prostate cancer patients. Gleason growth patterns are defined by their histological features on 4- to 5-µm cross sections, and little is known about their three-dimensional architecture. Our objective was to characterize the three-dimensional architecture of prostate cancer growth patterns. Intact tissue punches (*n* = 46) of representative Gleason growth patterns from radical prostatectomy specimens were fluorescently stained with antibodies targeting Keratin 8/18 and Keratin 5 for the detection of luminal and basal epithelial cells, respectively. Punches were optically cleared in benzyl alcohol–benzyl benzoate and imaged using a confocal laser scanning microscope up to a depth of 500 µm. Gleason pattern 3, poorly formed pattern 4, and cords pattern 5 all formed a continuum of interconnecting tubules in which the diameter of the structures and the lumen size decreased with higher grades. In fused pattern 4, the interconnections between the tubules were markedly closer together. In these patterns, all tumor cells were in direct contact with the surrounding stroma. In contrast, cribriform Gleason pattern 4 and solid pattern 5 demonstrated a three-dimensional continuum of contiguous tumor cells, in which the vast majority of cells had no contact with the surrounding stroma. Transitions between cribriform pattern 4 and solid pattern 5 were seen. There was a decrease in the number and size of intercellular lumens from cribriform to solid growth pattern. Glomeruloid pattern 4 formed an intermediate structure consisting of a tubular network with intraluminal epithelial protrusions close to the tubule splitting points. In conclusion, three-dimensional microscopy revealed two major architectural subgroups of prostate cancer growth patterns: (1) a tubular interconnecting network including Gleason pattern 3, poorly formed and fused Gleason pattern 4, and cords Gleason pattern 5, and (2) serpentine contiguous epithelial proliferations including cribriform Gleason pattern 4 and solid Gleason pattern 5.

## Introduction

The Gleason score is one of the most important parameters for therapeutic decision-making in men with prostate cancer and is entirely based on tumor growth patterns [1, 2]. Tumor heterogeneity is recognized by adding the two most common Gleason patterns in radical prostatectomy specimens. Gleason pattern 1, 2, and 3 prostate cancers are composed of well-delineated malignant glands, and the distinction of these patterns is putatively of no clinical significance [3]. Gleason pattern 4 tumors consist of poorly formed, fused, cribriform, or glomeruloid structures. Tumor growth in cords, single cells or solid fields, or the presence of comedonecrosis, characterizes Gleason pattern 5. Whereas men with Gleason score 6 (ISUP group 1) prostate cancer are often eligible for surveillance, active treatment is usually offered to patients with Gleason score ≥ 7 (ISUP group ≥ 2) [4].

Although individual growth patterns within Gleason patterns 4 and 5 are not routinely mentioned in pathology reports, numerous studies have demonstrated poorer outcomes when cribriform growth is present [5–9]. Cribriform growth in radical prostatectomies and diagnostic biopsies has been associated with more post-operative biochemical recurrence and disease-specific death in International Society of Urological Pathology (ISUP) group ≥ 2 prostate cancer patients [7]. On the other hand, ISUP group 2 patients with glomeruloid architecture may have a better outcome than those without this pattern [8]. Consideration of individual growth patterns may therefore have added value in the therapeutic stratification of ISUP group 2 prostate cancer patients.

A major limitation of the Gleason grading system is the substantial inter-observer variability [10–12]. Egevad et al. found that in a group of 337 European pathologists, only 56% agreement was achieved between expert consensus and participants’ score [13]. Inter-observer variability in Gleason grading occurs predominantly in the assessment of poorly formed and fused growth patterns [5, 7, 8, 12, 14]. In particular, small glands with sporadic lumen formation may be interpreted as tangentially sectioned Gleason pattern 3, poorly formed pattern 4, or cords pattern 5. Inter-observer variability significantly affects clinical decision-making since the distinction of ISUP group 1 prostate cancer from higher grades is an important threshold for active surveillance and treatment [4].

Diagnostic criteria for the histopathological grading of prostate cancer are entirely based on tumor features of routine 4- to 5-µm tissue sections. Very little is known about the underlying three-dimensional architecture of Gleason growth patterns. Serial sectioning and scanning of many tissue sections have given some insight into the three-dimensional tumor architecture; however, this is costly, time-consuming, and susceptible to artifacts. In contrast, optical tissue clearing allows for the sensitive fluorescent imaging of whole-tissue specimens without physical sectioning [15–18]. We have already demonstrated the feasibility of this technique for three-dimensional visualization of formalin-fixed, paraffin-embedded prostate tissues up to a depth of 800 µm [19]. The objective of the current study was to characterize and provide a comprehensive overview of the three-dimensional architecture of prostate cancer growth patterns.

## Materials and methods

### Case selection

Archival formalin-fixed, paraffin-embedded radical prostatectomy specimens from patients who had undergone radical prostatectomy for prostate cancer at the Erasmus Medical Center between 2012 and 2017 were included. Specimens were fixed in neutral-buffered formalin, transversely cut into 4-mm slices, and entirely embedded for histopathologic evaluation. The mean age at operation was 66 years (SD 6.8 years). Regions of interest for three-dimensional imaging were indicated on hematoxylin and eosin-stained slides by a urogenital pathologist. In total, 46 tumor areas from 35 patients were selected for analysis, including Gleason pattern 3 (*n* = 8), poorly formed (*n* = 6), fused (*n* = 6), glomeruloid (*n* = 10) and cribriform (*n* = 6) Gleason pattern 4, and cords (*n* = 7) and solid-fields (*n* = 3) Gleason pattern 5. Three normal peripheral zone areas were included to serve as a reference. The use of tissue samples for scientific purposes was approved by the institutional Medical Research Ethics Committee (MEC-2011-295, MEC-2011-296) and was in accordance with the “Code for Proper Secondary Use of Human Tissue in The Netherlands” as developed by the Dutch Federation of Medical Scientific Societies (FMWV, version 2002, update 2011).

### Immunofluorescent staining and optical clearing

Tissue punches from the areas with the selected growth patterns were taken from the corresponding paraffin blocks using a 500-µm diameter needle (Estigen Tissue Scuebcem, Tartu, Estonia) resulting in 3- to 4-mm-long cylindrical tissue cores with a diameter of 500 µm. Immunofluorescent staining and optical clearing were carried out according to an adapted iDISCO protocol as described previously (Supplementary table 1) [19, 20]. Briefly, punches were dewaxed, after which auto-fluorescence was blocked overnight. Subsequently, the punches were gradually rehydrated and incubated with primary Keratin 5 and Keratin 8/18 antibodies (1:150; EP1601Y; Abcam, Cambridge, UK and 1:75; MS-743; Immunologic, Duiven, The Netherlands) and secondary fluorescent Alexa-514- and Alexa-647-labeled antibodies (1:200; Life Technologies, Bleiswijk, The Netherlands). In order to visualize the subtle connective tissue cores within fused Gleason pattern 4, these samples were additionally stained with Fibronectin (FN1; 1:50; ab2413; Abcam, Cambridge, United Kingdom) and secondary Alexa-647 antibodies. The tissue was again dehydrated in methanol and subsequently optically cleared in benzyl alcohol–benzyl benzoate. Samples were then stored at 4 °C in the dark until imaging.

### Sample imaging

Fluorescently stained punches were imaged with an upright Leica SP5 confocal microscope equipped with a 1.95-mm working distance 20 × NA1.0 APO water dipping objective (Leica Microsystems GmbH, Wetzlar, Germany). Two-dimensional *Z*-stack images were recorded using a 488 nm Argon and a 633 nm HeNe laser with a 0.72 × 0.72 µm pixel size and 1–3 µm step size, resulting in 300–600 images per sample. Huygens Professional software (SVI, Hilversum, The Netherlands) with a theoretical point-spread function was used for de-convolution of the *Z* stacks, whereas three-dimensional rendering and image measurements were performed with Fiji (ImageJ 1.49s) and Amira (version 5.5.0; ThermoFisher Scientific, Waltham, USA) software [21]. *Z* stacks were loaded in Amira, after which we applied combined surface and volume rendering with standard settings. The total size of the *Z* stacks and three-dimensional renderings was 739 by 739 µm with a depth of 500 µm. Reference hematoxylin and eosin slides were positioned at a vertical side of the three-dimensional renderings, but were not directly continuous with the depicted areas in each case, depending on the site of imaging in the 3- to 4-mm-long cylindrical core.

### Pathological evaluation and statistical analysis

Both consecutive *Z* stacks and three-dimensionally rendered images were investigated. Pre-existent benign prostate glandular structures were identified by Keratin 8/18-positive luminal cells surrounded by Keratin 5-positive basal cells. Prostate cancer structures were recognized by architecturally disorganized Keratin 8/18-positive epithelial structures without a basal cell layer. Tubular blind-ending tips were identified by a detailed analysis of both *Z* stacks and three-dimensional renderings, and could be well distinguished from the transversely sectioned tubules at the border of the tissue samples. In each of the tissue specimens, the outer edges of the epithelial structures were measured in 3–5 consecutive two-dimensional slides per image and 3–10 individual epithelial structures per slide, depending on the growth pattern. This resulted in 12–50 measurements per tissue sample. Statistics were performed with a Student’s *t* test using the Statistical Package for Social Sciences (SPSS, version 24; IBM, Chicago, USA).

## Results

### Benign epithelial glands

**Fig. 1:**
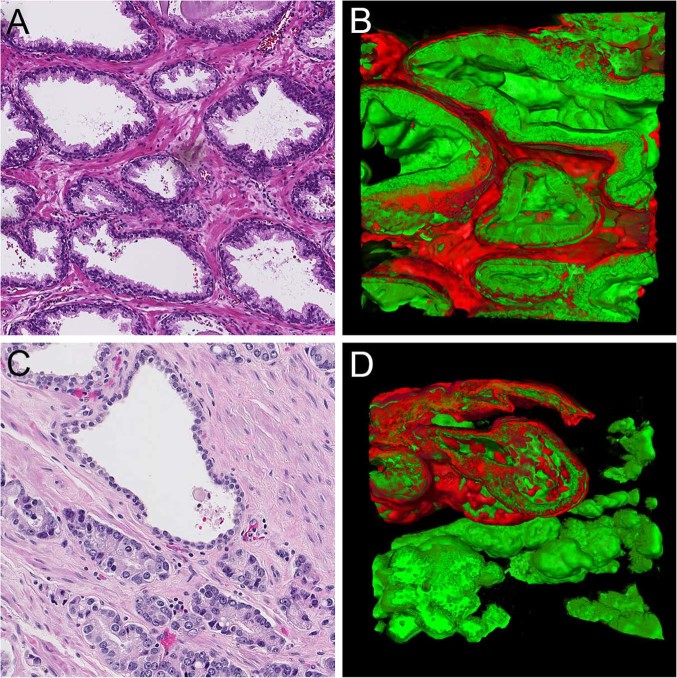
Peripheral zone **a** hematoxylin and eosin slide and **b** three-dimensional rendering, showing interconnecting saccules of variable width containing intraluminal papillary protrusions and surrounded by a continuous Keratin 5-positive basal cell layer (red). **c** Hematoxylin and eosin slide and **d** three-dimensional rendering of pre-existent benign glands surrounded by basal cells (upper left) and irregular malignant epithelial structures without a basal cell layer (lower). Original magnifications 20 × ; green, Keratin 8/18 and red, Keratin 5 immunostaining in three-dimensional renderings

Benign peripheral zone glands had an acinar organization composed of interconnecting saccules with variable intraluminal papillary protrusions. Benign glands were composed of an inner Keratin 8/18-positive luminal cell layer and an outer flat Keratin 5-positive layer of basal cells (Fig. 1a, b; Supplementary Video 1). In all cases, malignant epithelial structures could easily be distinguished from pre-existent benign glands by their architectural organization and lack of basal cells (Fig. 1c, d).

### Gleason pattern 3

**Fig. 2:**
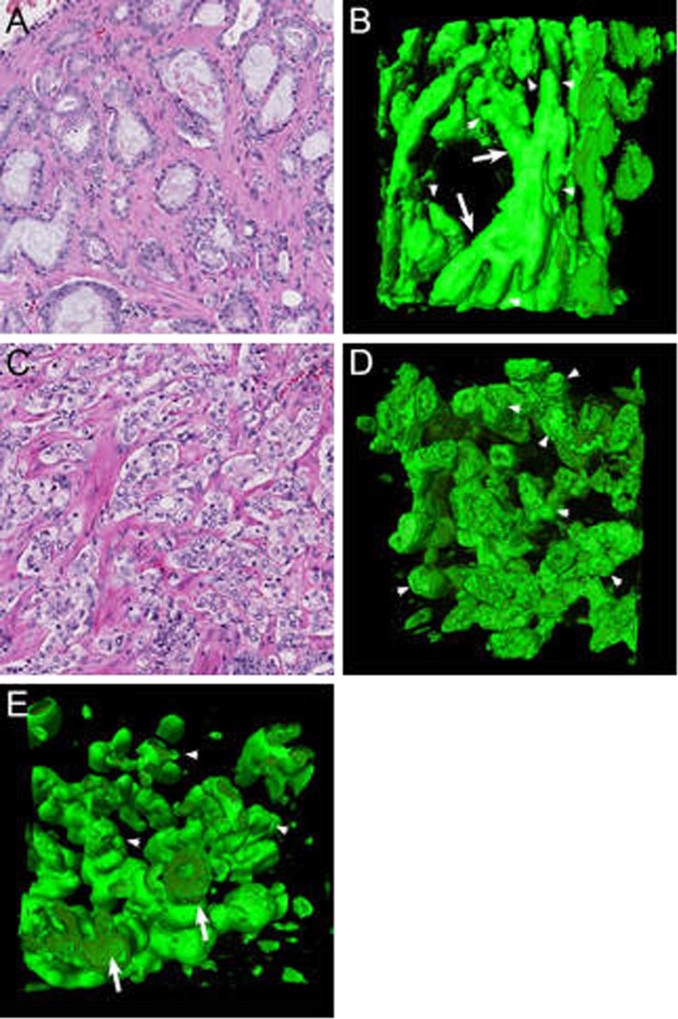
Gleason pattern 3 **a** hematoxylin and eosin slide and **b** three-dimensional rendering showing a tubular network with interconnections (arrows) and blind endings (arrowheads). Poorly formed Gleason pattern 4 **c** hematoxylin and eosin slide and **d** three-dimensional rendering of small-sized interconnecting tubules with blind endings (arrowheads). **e** Well-delineated Gleason pattern 3 tubules (arrows) were directly connected to and were continuous with poorly formed Gleason pattern 4 structures (arrowheads). Original magnifications 20 × ; green, Keratin 8/18 and red, Keratin 5 (no basal cells present) immunostaining in three-dimensional renderings

Gleason pattern 3 prostate cancer was composed of round to slightly oval, well-delineated curving tubules with a mean diameter of 45 µm (SD 12 µm) with regular interconnections (Fig. 2a, b; Supplementary Video 2). All malignant epithelial cells had contact with surrounding stroma. We did not find any specific tubular orientation along the cranial–caudal, transverse, or sagittal axis of the prostate. Blind-ending tubules were present sporadically and showed no specific location within the tubular network.

### Gleason pattern 4

#### Poorly formed glands

Poorly formed Gleason pattern 4 glands were represented by small round tubules with a significantly smaller average diameter than Gleason pattern 3 tubules (24 µm, SD 7 µm; *p* < 0.001). Poorly formed glands showed more frequent interconnections and blind endings than Gleason pattern 3 tubules (Fig. 2c, d). Regularly, we observed transitions between poorly formed Gleason pattern 4 glands and Gleason pattern 3 tubules (Fig. 2e; Supplementary Video 3).

#### Fused glands

**Fig. 3:**
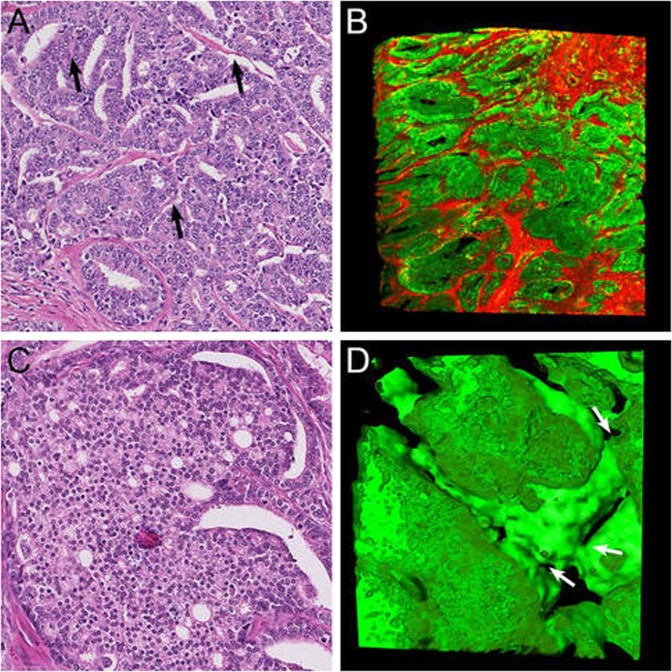
Fused Gleason pattern 4 **a** hematoxylin and eosin slide with subtle fibrovascular cores (arrows) and **b** three-dimensional reconstruction revealing frequently interconnecting tubules. Cribriform Gleason pattern 4 **c** hematoxylin and eosin slide and **d** three-dimensional rendering of cribriform fields displaying contiguous epithelial cells with spherical and ellipsoid intercellular lumens. The majority of tumor cells do not contact with the surrounding stroma. The cribriform areas formed serpentine structures with variably sized interconnections (arrows). Original magnifications 20 × ; green, Keratin 8/18 and red, Fibronectin (**b**) immunostaining in three-dimensional renderings

Fused Gleason pattern 4 glands consisted of round to oval tubules with a diameter of 68 µm (SD 18 µm), slightly larger than that of Gleason pattern 3 tubules (*p* < 0.001). The hallmark of the fused pattern was the presence of abundant interconnections between the tubules, which occurred markedly closer together than in the aforementioned patterns (Fig. 3a, b). On hematoxylin and eosin slides, fused Gleason pattern 4 can closely resemble the cribriform architecture [12, 13]. Here three-dimensional microscopy, however, revealed subtle intervening fibrovascular tissues in between and around all malignant tubules, revealing that all malignant cells had contact with the surrounding stroma (Supplementary Video 4).

#### Cribriform fields

Cribriform Gleason pattern 4 was characterized by fields of contiguous epithelial tumor cells with a mean diameter of 151 µm (SD 68 µm). The vast majority of tumor cells did not have any contact with the surrounding stroma, in contrast with the patterns described previously. Three dimensionally, this pattern showed a variable number of spherical, ellipsoid, slit-like, or irregular interconnecting intercellular lumens (Fig. 3c, d). Adjacent cribriform fields with intervening stroma on hematoxylin and eosin slides represented continuously curving irregular serpentine structures on three-dimensional renderings (Supplementary Video 5). We did not observe any transition between cribriform fields and aforementioned tubular structures in our cohort.

#### Glomeruloid glands

**Fig. 4:**
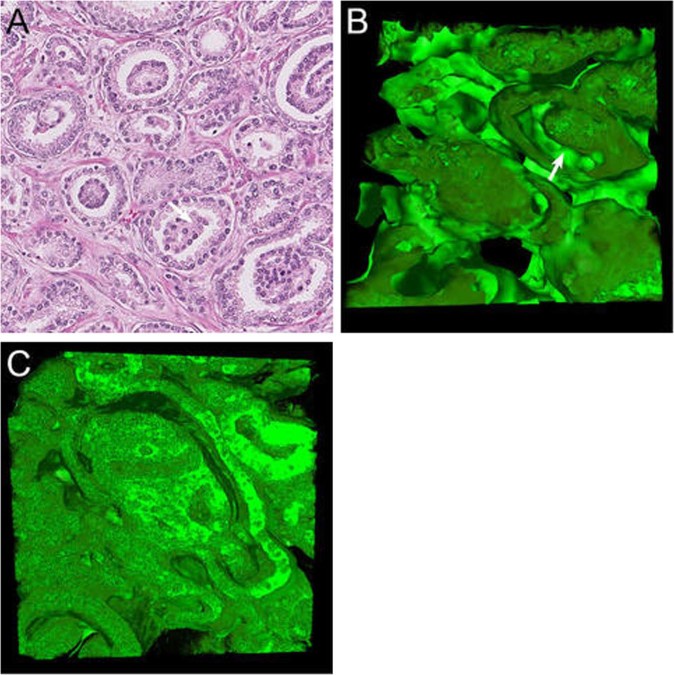
Glomeruloid Gleason pattern 4 **a** hematoxylin and eosin slide and **b** three-dimensional rendering of small glomeruloid protrusions, consisting of malignant epithelial cells in contact with the epithelial tubular lining at one side of the tubule (arrow). **c** Large glomeruloid protrusions with intercellular lumens. While this structure resembles the cribriform architecture, the glomeruloid protrusion only makes contact with one side of the tubule. Original magnifications 20 × , green, Keratin 8/18 and red, Keratin 5 (no basal cells present) immunostaining in three-dimensional renderings

In two-dimensional cross sections, glomeruloid Gleason pattern 4 structures resemble renal glomeruli and are characterized by dilated glands with round protrusions of malignant epithelial cells (Fig. 4a, b). On three-dimensional renderings, these glomeruloid structures were present within an interconnecting network of tubules, which had a mean diameter of 65 µm (SD 19 µm), reminiscent of Gleason pattern 3 glands, but with larger tubule diameters (*p* < 0.001). Two different glomeruloid structures could be distinguished using three-dimensional microscopy. The first type was nodular epithelial glomeruloid proliferations, which connected to the tumor cells lining the tubule on one side, but did not make any contact with the tubular lining on the opposite side or the surrounding stroma. These protrusions often occurred at tubular branching points (Supplementary Video 6). The second type showed the presence of subtle fibrovascular cores on the hematoxylin and eosin slides, representing clusters of markedly curved tubules in three dimensions. All the tumor cells within this glomeruloid variant made contact with the surrounding stroma. When glomeruloid structures are larger, distinction between glomeruloid and cribriform growth patterns on hematoxylin and eosin slides can be challenging (Fig. 4c) [12]. These larger cribriform-like structures grew similar to the first type of glomeruloid pattern in three-dimensional renderings. We did not observe any continuity between glomeruloid structures and the cribriform pattern.

### Gleason pattern 5

#### Cords and single cells

**Fig. 5:**
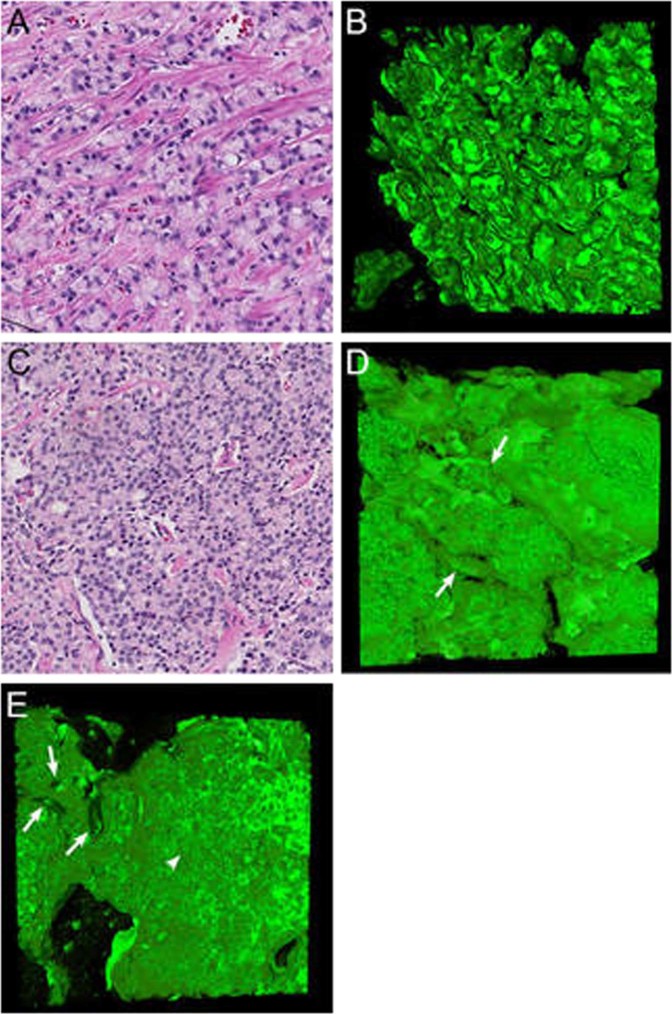
Gleason pattern 5 cords **a** hematoxylin and eosin slide and **b** three-dimensional rendering with interconnecting cords consisting of one or two tumor cells without lumens. Solid Gleason pattern 5 **c** hematoxylin and eosin slide and **d** three-dimensional rendering showing solid structures with variably sized interconnections (arrows). **e** Transition from cribriform Gleason pattern 4 (left side) with multiple lumens (arrows) to solid Gleason pattern 5 (right side) lacking lumen formation (arrowhead). Original magnifications 20 × ; green, Keratin 8/18 and red, Keratin 5 (no basal cells present) immunostaining in three-dimensional renderings

On hematoxylin and eosin slides, Gleason pattern 5 cords consist of one- or two-layered strands of cells without distinctive lumens (Fig. 5a). In three dimensions, cords and single-cell structures formed a continuous meshwork consisting of one or two cell layers with extensive branching and interconnections. The average diameter of these cords was 15 µm (SD 7 µm; Fig. 5b), significantly smaller than poorly formed Gleason pattern 4 (*p* < 0.001). Small intercellular lumens were observed at deeper levels of the cord pattern in the *Z* stack, indicating repetitive subtle transitions from Gleason pattern 4 poorly formed tubules to Gleason pattern 5 cords (Supplementary Video 7).

#### Solid fields

Solid-fields Gleason pattern 5 are represented on hematoxylin and eosin slides as round or irregularly formed areas composed of tumor cells without intercellular lumens. Most cells do not make any contact with the surrounding stroma (Fig. 5c). Three-dimensional reconstruction has revealed that the solid fields represented round to ellipsoid irregular serpentine structures with an average diameter of 185 µm (SD 78 µm), interconnecting and varying in width (Fig. 5d; Supplementary Video 8). While intercellular lumens were inconspicuous on hematoxylin and eosin slides, the *Z* stacks often showed small, round, and ellipsoid lumens, reminiscent of cribriform Gleason pattern 4 at deeper levels, indicating a transition between these patterns. In this cohort, we did not find any transition between solid-fields Gleason pattern 5 and tubular growth patterns.

## Discussion

In the current study, we provided a comprehensive overview of the three-dimensional architecture of prostate cancer growth patterns and revealed two architecturally different growth pattern subgroups. The first subgroup consisted of a tubular network in which the vast majority of the tumor cells made direct contact with the surrounding stroma. We demonstrated that Gleason pattern 3 glands formed a network of regularly interconnecting tubules. In poorly formed Gleason pattern 4, the network consisted of smaller sized tubules, and in fused Gleason pattern 4, the tubules showed frequent and closely spaced interconnections. In glomeruloid Gleason pattern 4, intraluminal epithelial protrusions occurred within the tubular network, close to tubular branching points. Cords Gleason pattern 5 represented a network structure with frequent interconnections without lumens. The second subgroup was characterized by contiguous tumor-cell proliferations in which the vast majority of tumor cells did not make any contact with the surrounding stroma, consisting of cribriform Gleason pattern 4 and solid pattern 5. This subgroup represented irregular serpentine structures of contiguous tumor cells with a decrease in frequency and size of intercellular lumens from cribriform to solid pattern.

An important advantage of three-dimensional imaging is the visualization of morphological transitions and continuity of growth patterns, which can, generally, not be appreciated in routine two-dimensional sections. Until now, only a few studies have aimed to reconstruct prostate cancer growth patterns in three dimensions, using sectioning and alignment of numerous sequential slides [15, 22–26]. For instance, Boag et al. used this method on five different cases to generate three-dimensional renderings, showing interconnections between Gleason pattern 3 and pattern 4 glands [26]. Similarly, Tolkach et al. demonstrated the continuity between Gleason pattern 3 and pattern 4 after the serial sectioning and three-dimensional rendering of one ISUP group 2 case [25]. Apart from being laborious, the stacking of sequentially cut slides is prone to tissue malformation and registration artifacts. In contrast, fluorescent staining, tissue clearing, and long-distance confocal scanning microscopy are performed on intact tissue samples without tissue sectioning, thus preventing alignment artifacts. It is also less laborious, although specialized microscopic equipment is required [19]. Our finding of the three-dimensional continuity between Gleason patterns 3 and 4 is in line with the aforementioned studies, even though different methods were applied.

The most important observation of this study was that we identified two architecturally different growth pattern subgroups. Firstly, there are interconnecting tubular structures, consisting of Gleason pattern 3, poorly formed and fused Gleason pattern 4, and cords Gleason pattern 5. These patterns have variable tubule diameters, interconnection frequencies, and lumen formations, but the vast majority of the tumor cells make direct contact with the surrounding stroma. In our cohort, we observed frequent transitions between these growth patterns. While poorly formed glands had smaller sized tubules than Gleason pattern 3, no clear cut-off could be made between these patterns with respect to tubule diameter, number of interconnections, or luminal size. Similarly, three-dimensional spatial transitions between poorly formed Gleason pattern 4 and Gleason pattern 5 cords made the strict delineation of these patterns impossible. The increased number of interconnections in fused Gleason pattern 4 was only arbitrarily distinguished from branching Gleason pattern 3 tubules. The three-dimensional continuity of these patterns is reflected by the substantial inter-observer variability in daily pathology practice. Distinguishing, on one hand, tangentially sectioned Gleason pattern 3 glands from poorly formed and fused Gleason pattern 4 glands, and, on the other hand, poorly formed Gleason pattern 4 glands from Gleason pattern 5 cords on hematoxylin and eosin slides is the principal area of difficulty [12, 14, 27, 28]. Secondly, there are serpentine compact irregular epithelial proliferations, consisting of cribriform Gleason pattern 4 and solid Gleason pattern 5, with decreasing inter-epithelial lumen sizes and frequencies. Both patterns show in common that the vast majority of tumor cells are contiguous and do not make contact with the surrounding stroma. Although we did not include comedonecrosis in this study, routine diagnostic slides reveal that comedonecrosis predominantly occurs in a background of cribriform and solid structures. We found transitions between cribriform Gleason pattern 4 and solid Gleason pattern 5 but did not observe any transition between these patterns and the aforementioned tubular growth pattern subgroup.

The inter-observer agreement of cribriform Gleason pattern 4 is excellent. The only variability there occurs in the distinction between complex fused and large glomeruloid patterns [12]. Of interest, our three-dimensional images showed that although complex fused Gleason pattern 4 glands might resemble cribriform Gleason grade 4 structures on hematoxylin and eosin slides, scattered subtle intra-lesion fibrovascular cores were present in complex fused Gleason pattern 4 glands as a distinguishing feature. On hematoxylin and eosin slides, glomeruloid growth morphologically represents an intermediate pattern between tubular and cribriform growths. While some glomeruloid structures with subtle fibrovascular cores actually closely resembled fused Gleason pattern 4 glands in three dimensions, most glomeruloid structures did not contain intra-lesional connective tissue. Based on morphological resemblance and frequent coexistence, Lotan and Epstein hypothesized that glomeruloid pattern is a precursor of cribriform growth [29]. However, Choy et al. found that ISUP group 2 and 3 prostate cancer patients with the glomeruloid pattern had significantly lower biochemical recurrence rates than those with cribriform growth [5]. Our three-dimensional reconstructions did not reveal any continuity between glomeruloid and cribriform structures. Therefore, the clinical relevance of the glomeruloid pattern and its place as a putative precursor of cribriform growth remain to be established.

Various studies have shown that prostate cancer patients with an ISUP group 2 tumor showing cribriform Gleason pattern 4 have a worse outcome than patients without this pattern. Kweldam et al. found that patients with an ISUP group 2 tumor without cribriform growth on biopsy had similar metastasis-free survival and biochemical recurrence rates as patients with ISUP group 1 prostate cancer [7, 8]. The adverse outcome related to cribriform growth was also present in men with ISUP group > 2 prostate cancer [8, 30]. A putative explanation for the worse outcome of patients with cribriform growth pattern is the fact that cribriform architecture is associated with genomic instability, while non-cribriform Gleason pattern 4 is genomically indistinguishable from Gleason pattern 3 [31–33]. These clinical and molecular observations are in line with the two architectural subgroup hypotheses as raised in the current study. To the best of our knowledge, there are, as yet, no studies on the clinical relevance of Gleason 5 growth patterns. Investigation into the prognostic value of these individual Gleason grade 5 patterns is essential for future tumor grading and understanding. Based on our current findings, we hypothesize that solid Gleason pattern 5 is likely to be associated with a worse outcome than cords or single-cells Gleason pattern 5.

This study represents the first comprehensive three-dimensional characterization of relevant prostate cancer growth patterns. Optical clearing of intact samples allows the visualization and investigation of tumor growth patterns without the need of sectioning and alignment of numerous consecutive tissue slides. We studied the most common prostate cancer growth patterns, but did not include the full spectrum of growth patterns and variants that can be encountered in daily practice [34, 35]. While we selected unambiguous cases of the Gleason growth patterns, inter-observer variability might still exist, for instance, in labeling, as either poorly formed or fused Gleason pattern 4 [12, 13]. Another disadvantage of three-dimensional pathology is the descriptive terminology that is used for reporting. Three-dimensional imaging of prostate cancer, however, allows for objective geometrical modeling in a three-dimensional matrix. Finally, while we identified two three-dimensional growth pattern subgroups with glomeruloid architecture as an intermediate structure, the differences between these subgroups on the clinical and molecular level remain to be investigated.

In conclusion, this study gives a comprehensive overview of the three-dimensional architecture of prostate cancer growth patterns. We show the existence of two major architectural growth pattern subgroups: (1) a tubular interconnecting network of tumor cells in direct contact with adjacent stroma, with a variable gland and lumen size, including Gleason pattern 3, poorly formed and fused Gleason pattern 4, and cords Gleason pattern 5, and (2) serpentine contiguous epithelial proliferations in which the majority of tumor cells do not make contact with the adjacent stroma and with variable inter-epithelial lumen frequency, including cribriform Gleason pattern 4 and solid Gleason pattern 5. An insight into tumor-growth patterns facilitates the comprehension of prostate cancer behavior and biology beyond the current Gleason grading.

## Supplementary information

Supplementary Video 1

Supplementary Video 2

Supplementary Video 3

Supplementary Video 4

Supplementary Video 5

Supplementary Video 6

Supplementary Video 7

Supplementary Video 8

Supplementary Video Legends

Supplementary Table 1
