# Supplementary material for: Three-dimensional analysis reveals two major architectural subgroups of prostate cancer growth patterns
Source: Mod Pathol. 2019 Feb 8;32(7):1032–41. doi: 10.1038/s41379-019-0221-0 (PMC6760644; doi:10.1038/s41379-019-0221-0)
Supplement: Supplementary file 9 — Supplementary Video Legends [file 41379_2019_221_MOESM9_ESM.docx]

**Supplementary Table and Video legends**

**Supplementary Table 1.** Protocol for fluorescent staining and clearing of fresh and formalin-fixed paraffin-embedded specimens.

**Supplementary Video 1.** Three-dimensional rendering of peripheral zone with large interconnecting saccules of varying width containing intraluminal papillary protrusions and a continuous layer of basal cells. Original magnification 20x, green Keratin 8/18 and red Keratin 5 immunostaining.

**Supplementary Video 2.** Three-dimensional rendering of Gleason pattern 3, showing interconnecting and blind-ending tubules. Original magnification 20x, green Keratin 8/18 and red Keratin 5 immunostaining.

**Supplementary Video 3.** Three-dimensional rendering of poorly formed Gleason pattern 4 displaying frequently interconnecting small-sized tubules, in continuity with Gleason patterns 3. Original magnification 20x, green Keratin 8/18 and red Keratin 5 immunostaining.

**Supplementary Video 4.** Z-stack of fused Gleason pattern 4 with frequently interconnecting tubules with intervening stroma. Original magnification 20x, green Keratin 8/18 and red Fibronectin immunostaining.

**Supplementary Video 5.** Z-stack of cribriform Gleason pattern 4 with serpentine and interconnecting fields of contiguous malignant epithelial cells. The majority of tumor cells do not make contact with the surrounding stroma. Intercellular lumens are spherical, ellipsoid or irregular shaped. Original magnification 20x, green Keratin 8/18 and red Keratin 5 immunostaining.

**Supplementary Video 6.** Z-stack of glomeruloid Gleason pattern 4, showing glomeruloid protrusions at tubule splitting points. Original magnification 20x, green Keratin 8/18 and red Keratin 5 immunostaining.

**Supplementary Video 7.** Z-stack of Gleason pattern 5 cords consisting of one- to two-layered strands of epithelial tumor cells. While Gleason patterns 5 cords did not show lumens, transitions can frequently be observed between this pattern and poorly formed Gleason pattern 4 with intercellular lumens. Note that the intracellular small round dark areas mostly represent nuclei, while poorly formed glands are identified by lumens between two or more individual epithelial tumor cells. Original magnification 20x, green Keratin 8/18 and red Keratin 5 immunostaining.

**Supplementary Video 8.** Z-stack of solid Gleason pattern 5 consisting of serpentine interconnecting fields of variable width, composed of contiguous malignant epithelial cells. While no intercellular lumens were present in solid Gleason pattern 5, sporadic intercellular lumen formation can be observed at deeper levels of the Z-stack, reminiscent of cribriform Gleason pattern 4. Original magnification 20x, green Keratin 8/18 immunostaining.
